# Cannabis-Related Disorders Are Associated with Increased Early Postoperative Opioid Prescriptions and Delayed Emergency Department Visits Following Open Carpal Tunnel Release

**DOI:** 10.1177/15589447241284788

**Published:** 2024-10-21

**Authors:** Kirstin A. Humble, Sohrab K. Vatsia, Peter F. Monahan, Kenneth F. Taylor

**Affiliations:** 1 Department of Orthopedics and Rehabilitation, Penn State Health Milton S. Hershey Medical Center, Hershey, PA, USA; 2 Penn State College of Medicine, Hershey, PA, USA

**Keywords:** cannabis, carpal tunnel release, database, pain management

## Abstract

**Background:**

The effect of cannabis on pain management following open carpal tunnel release (CTR) surgery is unknown. The purpose of this study is to compare outcomes for patients with cannabis-related disorder (CRD) undergoing open CTR to a propensity-matched cohort of patients without CRD (no cannabis-related disorder [NCRD]).

**Methods:**

The TriNetX Research Network was queried to identify patients undergoing primary open CTR between January 2010 and December 2022. Patients with CRD were propensity matched to a NCRD cohort in a 1:1 ratio based on 7 characteristics. Rates of postoperative opioid prescriptions, emergency department (ED) services, and outpatient appointments were reported at 0-2, 2-6, and 6-12 weeks postoperatively.

**Results:**

A total of 925 CRD patients were propensity matched to 925 NCRD patients undergoing open CTR. Within 0-2 weeks postoperatively, CRD patients received significantly greater rates of opioid prescriptions compared to NCRD patients (30.9% and 25.6%, *P* = .011). No cannabis-related disorder (NCRD) patients presented for outpatient follow-up at significantly higher rates than CRD patients within 6 weeks postoperatively. CRD patients presented to the ED at significantly higher rates between 6 and 12 weeks postoperatively (11.0% vs. 8.0%, respectively, *P* = .027).

**Conclusions:**

Cannabis-related disorder (CRD) is associated with lower rates of outpatient follow-up but higher rates of postoperative opioid prescriptions and ED presentations following open CTR compared to a propensity-matched cohort of NCRD patients.

**Level of Evidence:**

Cohort Study; Level III.

## Introduction

Cannabis is one of the most commonly used recreational psychoactive substances in the United States with an estimated 52.4 million persons age 12 or older reporting cannabis usage in 2021.^1^ Correspondingly, national cannabis legalization efforts have correlated with increased patient-reported cannabis usage, largely due to its analgesic and sedative properties.^2–4^ Given the growing economic and mortality burden associated with the ongoing opioid epidemic, cannabis has been proposed as an adjunct therapeutic option for acute and chronic pain—particularly within the orthopedic perioperative setting.^5–8^ Despite its potential as a pain management alternative, cannabis has been associated with a spectrum of comorbid cannabis-related disorder (CRD), which have been linked to increased postoperative pain in patients undergoing orthopedic surgery.^9,10^

Open carpal tunnel release (CTR) for carpal tunnel syndrome is one of the most frequently performed procedures within the scope of hand surgery, and pain management remains a persistent challenge for hand surgeons.^11,12^ There is a dearth of evidence in the existing literature examining the characteristics and postoperative outcomes of patients with CRD undergoing primary open CTR. In consideration of the prevalence of cannabis use as well as the commonplace nature of open CTR, investigation of this patient population will undoubtedly afford greater insight into the underlying effects of CRD and its postoperative clinical and economic outcomes.

Therefore, this study sought to interrogate rates of early postoperative opioid usage and healthcare utilization in patients with CRD undergoing open CTR to better inform the postoperative management of carpal tunnel syndrome. We hypothesize that CRD patients would have increased need for opioid prescription and healthcare utilization compared to a propensity-matched cohort of no cannabis-related disorder (NCRD) patients.

## Methods

### Data Query

The TriNetX Research Network was queried using a combination of International Classification of Disease-10 (ICD-10) and Current Procedural Terminology (CPT) codes. Patients were included in this study if they underwent primary open CTR surgery (CPT 64721) with a concomitant diagnosis of carpal tunnel syndrome (ICD-10 G56.0).^13^ All patients were at least 18 years old and underwent surgery between January 1, 2010 and December 31, 2022. Patients were excluded from our analysis if they underwent prior open CTR surgery or if they underwent endoscopic CTR. Patients were divided into 1 of 2 cohorts: patients with a diagnosis of CRD (ICD-10 F12) within 1 year prior to surgery and patient who did not (NCRD). Cannabis-related disorder (CRD) is defined as “cannabis abuse,” “cannabis dependence,” and “cannabis use, unspecified.” A complete list of codes to identify patients for this study can be found in Supplement 1.

### About the TriNetX Research Network

The TriNetX, LLC Research Network (Cambridge, Massachusetts) was queried on January 28, 2024. TriNetX aggregates data from several ambulatory, primary care, and pharmacy clearinghouse claims and offers several networks for research purposes. This study utilized the Research Network, which consists of more than 120 million patients across 85 health care systems. All data are deidentified and Health Insurance Portability and Accountability Act (HIPAA) compliant and, therefore, was exempt from institutional review board approval. There is a growing body of orthopedic literature using TriNetX.^14–19^

### Outcomes

Patient outcomes were analyzed within several time intervals following primary open CTR. Postoperative opioid prescriptions, emergency department (ED) visits, and global period outpatient appointments were recorded during the 0-2 weeks, 2-6 weeks, and 6-12 weeks following surgery. Opioid prescriptions were recorded collectively and by individual prescription using the Veterans Affairs (VA) and RxNorm classification system. Emergency department (ED) visits were defined using CPT codes related to utilization of ED services. Global period outpatient appointments were recorded using postoperative visits included in the patients’ surgical packages.

Outcomes were reported based on the percentage of patients with the outcome in question (rate), the number of prescriptions per patient, and percentage difference between cohorts. A list of all codes used to record these outcomes is listed in Supplement 1.

### Statistical Analysis

Comorbidities of CRD and NCRD cohorts were collected and analyzed using descriptive statistics. Mean, standard deviation, rate, and mean differences were used to report findings. Ultimately, CRD patients were propensity matched in a 1:1 ratio to NCRD patients based on female sex as reported in TriNetX, anxiety, stress-related and somatoform disorders, morbid obesity, white race, preoperative opioid prescriptions, and prior use of ED services. Statistical significance was set at *P* < .05.

## Results

### Cohort Demographics

The TriNetX Research Network identified 116 223 patients undergoing open CTR between January 2010 and December 2022. Of these 925 (0.8%) had a diagnosis of CRD within 1 year of surgery. Significant differences were present between preoperative comorbidities for CRD and NCRD cohorts. Patients with CRD were significantly younger (47.4 ± 12.9 vs. 56.8 ± 14.0, *P* < .0001), had higher rates of anxiety disorders (51.9% vs. 14.4%, *P* < .0001), and a higher history of ED services (58.1% vs. 17.4%, *P* < .0001) compared to the NCRD cohort among other comorbidities (Table 1).

**Table 1.** Matching Criteria of CRD and NCRD Patients Undergoing Primary Open Carpal Tunnel Release.

	Before matching			After matching		
	CRD	NCRD	*P*-value	CRD	NCRD	*P*-value
Patients	925	115 298		925	925	
Age at index	47.4 ± 12.9	56.8 ± 14.0	**<.001**	47.4 ± 12.9	47.4 ± 12.8	.976
White	593 (64.1)	82 796 (71.8)	**<.001**	593 (64.1)	596 (64.4)	.884
Female sex	465 (50.3)	66 026 (57.3)	**<.001**	465 (50.3)	471 (50.9)	.78
Anxiety disorders	480 (51.9)	16 551 (14.4)	**<.001**	480 (51.9)	480 (51.9)	>.999
Morbid obesity	123 (13.3)	7411 (6.4)	**<.001**	123 (13.3)	113 (12.2)	.486
ED services	537 (58.1)	20 045 (17.4)	**<.001**	537 (58.1)	539 (58.3)	.925
Opioid analgesics	695 (75.1)	46 045 (39.9)	**<.001**	695 (75.1)	698 (75.5)	.872

Values are presented as mean ± standard deviation or *n* (%). Statistical significance is denoted in bold if *P* < .05. CRD, cannabis-related disorders; NCRD, no cannabis-related disorders; ED, emergency department.

After propensity matching for 7 comorbidities, all 925 CRD patients were successfully propensity matched to 925 NCRD patients. Propensity matching successfully offset the significant differences in the initial cohorts to create more homogeneous cohorts. Cohorts were found to have similar rates of comorbidities for all matching criteria. These data are presented in Table 1.

### Postoperative Opioid Prescriptions

The CRD cohort undergoing open CTR received significantly higher rates of opioid prescriptions compared to NCRD patients within 2 weeks postoperatively (30.9% vs. 25.6%, respectively, *P* = .011). However, rates of opioid prescribing were similar between cohorts for the 2-6 week postoperative time interval as well as the 6-12 week postoperative interval (Table 2).

**Table 2.** Number of Patients Undergoing Open Carpal Tunnel Release Receiving Opioid Prescriptions by Specified Postoperative Time Interval for CRD and NCRD Cohorts.

	CRD	NCRD	*P*-value
Interval			
0-2 weeks	286 (30.9)	237 (25.6)	.**011**
2-6 weeks	249 (26.9)	225 (24.3)	.201
6-12 weeks	243 (26.3)	236 (25.5)	.710

All values are presented as *n* (%). Statistical significance is denoted in bold if *P* < .05. CRD, cannabis-related disorders; NCRD, no cannabis-related disorders.

Table 3 presents the average number of prescriptions received among the patients who received a postoperative prescription. Of the patients who received opioid prescriptions, CRD patients received a significantly greater number of prescriptions per patient for all time intervals. In the 6-12 week postoperative interval, CRD patients received an average of 2.59 ± 3.83 opioid prescriptions compared to NCRD patients who received 1.95 ± 1.58 prescriptions (*P* = .017).

**Table 3.** Average Number of Opioid Prescriptions Per Patient Prescribed for CRD and NCRD Cohort Undergoing Open Carpal Tunnel Release.

	CRD	NCRD	*P*-value
Interval			
0-2 weeks	2.3 ± 2.8	1.6 ± 1.3	.**0001**
2-6 weeks	2.3 ± 3.0	1.5 ± 1.0	.**001**
6-12 weeks	2.6 ± 3.8	2.0 ± 1.6	.**017**

Statistical significance is denoted in bold if *P* < .05. CRD, cannabis-related disorders; NCRD, no cannabis-related disorders.

When analyzing specific postoperative opioid medication prescriptions, CRD patients received significantly higher rates of hydromorphone (5.9% vs. 3.7%, respectively, *P* = .02) and oxycodone (16.8% vs. 11.1%, respectively, *P* < .0001) during the 0-2 week time interval postoperatively. Percentage difference between specific opioid medication prescriptions is presented in Table 4.

**Table 4.** Percentage Difference Between CRD and NCRD Patients Receiving Specific Opioid Medication Following Open Carpal Tunnel Release.

	0-2 weeks	2-6 weeks	6-12 weeks
Tramadol	0.9	1.5	0.6
Hydromorphone	**2.3**	−0.6	0.2
Hydrocodone	−0.1	1.6	−1.2
Fentanyl	1.6	0.6	1.5
Oxycodone	**5.6**	2.7	1.2
Other	1.8	0	1.2

Values are presented as percentage differences between CRD and NCRD cohorts. Bold values indicate statistical significance (*P* < .05). CRD, cannabis-related disorders; NCRD, no cannabis-related disorders.

### Medical Resource Utilization

A significantly higher percentage of patients with NCRD attended postoperative outpatient visits compared to CRD patients following open CTR at both the 0-2 week (41.6% vs. 36.5%, respectively, *P* = .025) and 2-6 week time interval (34.8% vs. 30.1%, respectively, *P* = .029). Patients in both cohorts attended a similar rate of outpatient postoperative appointments within the 6-12 week interval (*P* = .28).

Patients in both cohorts utilized similar rates of ED services within 6 weeks postoperatively. However, during the 6-12 week time interval, CRD patients utilized significantly higher rates of ED services (*P* = .03). All medical resource utilization data are presented in Table 5.

**Table 5.** Medical Resource Utilization for Propensity-Matched CRD and NCRD Cohorts Undergoing Open Carpal Tunnel Release.

	CRD	NCRD	*P*-value
Outpatient visits			
0-2 weeks	338 (36.5)	385 (41.6)	.**025**
2-6 weeks	278 (30.1)	322 (34.8)	.**029**
6-12 weeks	217 (23.5)	237 (25.6)	.28
Emergency department visits			
0-2 weeks	42 (4.5)	41 (4.4)	.911
2-6 weeks	68 (7.4)	53 (5.7)	.158
6-12 weeks	102 (11.0)	74 (8.0)	.**027**

All values are presented as *n* (%). Statistical significance is denoted in bold if *P* < .05. CRD, cannabis-related disorders; NCRD, no cannabis-related disorders.

## Discussions

With greater than 52 million Americans reporting cannabis usage in 2021, and its association with surgical complications, further investigation into this is necessary.^1,20,21^ The present study analyzed postoperative opioid prescribing and healthcare utilization for patients with CRD undergoing open CTR compared to patients without CRD.

We discovered that CRD patients undergoing open CTR surgery received higher numbers of opioid prescriptions at all time points postoperatively, compared to a matched NCRD population. This is consistent with the investigation performed by Dietz et al, which also demonstrated a higher rate of opioid consumption in patients with cannabis use, following cervical and lumbar spine fusions.^22^ The number of opioid prescriptions was highest in the immediate postoperative period. This too is consistent with literature, which does show higher postoperative opioid consumption in the immediate postoperative period.^23^ There were significantly higher rates of hydromorphone and oxycodone prescribing at this 0-2 week time period. This differential in prescribing practices also appears to be previously undescribed in the literature.

A significantly higher percentage of NCRD patients attended postoperative outpatient visits at the 0-2 week and 2-6 week time intervals. Cannabis use in patients has been associated with higher rates of missed clinic appointments in non-orthopedic literature, and appears to also be the case with our cohort of CRD patients.^24^ The rates of attendance at the 6-12 week time interval was similar for CRD and NCRD groups. As follow-up for a CTR after the 6-week period is not necessarily standard, these similar rates of appointment utilization could perhaps be attributed to patients with postoperative complications or concerns, requiring them to come into the office. Patients with CRD sought care in the ED at higher rates in the 6-12 week postoperative period. Further investigation would be necessary to determine if these visits were secondary to postoperative complications or concerns, or due to an unrelated issue.

There is limited literature analyzing CRD in patients undergoing open CTR; however, this study is not without limitations. First, TriNetX database is a deidentified and encrypted database that upholds HIPAA compliance. This limits the authors’ ability to ensure patient data is complete for the analysis performed. Second, ICD-10, CPT, and RxNorm codes were used to query patients and analyze data. There is an unintentional possibility of a coding or classification error that may affect our results. For example, we cannot confirm if the CRD cohort was using cannabis at the time of CTR because we stratified the cohorts based on the ICD-10 code F12. Third, we are unable to determine the chief complaint for which a patient used ED services, or what concerns they may have had during outpatient follow-up. It is possible to use TriNetX database to decipher these reasons, but this would require a separate query, which could be an area of future research. Lastly, although prescription rates and number of prescriptions per person were recorded, we are not able to establish more granular details such as the dosage or length of prescription.

## Conclusions

CRD is associated with lower rates of outpatient follow-up but higher rates of postoperative opioid prescriptions and ED presentations following open CTR compared to a propensity-matched cohort of NCRD patients.

## Supplemental Material

sj-docx-1-han-10.1177_15589447241284788 – Supplemental material for Cannabis-Related Disorders Are Associated with Increased Early Postoperative Opioid Prescriptions and Delayed Emergency Department Visits Following Open Carpal Tunnel Release Supplemental material, sj-docx-1-han-10.1177_15589447241284788 for Cannabis-Related Disorders Are Associated with Increased Early Postoperative Opioid Prescriptions and Delayed Emergency Department Visits Following Open Carpal Tunnel Release by Kirstin A. Humble, Sohrab K. Vatsia, Peter F. Monahan and Kenneth F. Taylor in HAND
