# Supplementary material for: Cannabis-Related Disorders Are Associated With Increased Early Postoperative Opioid Prescriptions and Delayed Emergency Department Visits Following Open Carpal Tunnel Release
Source: Hand (N Y). 2024 Oct 21;20(8):1232–6. doi: 10.1177/15589447241284788 (PMC11559794; doi:10.1177/15589447241284788)
Supplement: sj-docx-1-han-10.1177_15589447241284788 – Supplemental material for Cannabis-Related Disorders Are Associated with Increased Early Postoperative Opioid Prescriptions and Delayed Emergency Department Visits Following Open Carpal Tunnel Release [file sj-docx-1-han-10.1177_15589447241284788.docx]

| **Supplement 1** | | |
| --- | --- | --- |
| **Classification Codes for Data Query, Matching Criteria, and Outcomes** | | |
| Name | Code Type | Code |
| **Inclusion Criteria** |  |  |
| Neuroplasty and/or transposition; median nerve at carpal tunnel | CPT | 64721 |
| Carpal Tunnel Syndrome | ICD-10 | G56.0 |
| Cannabis Related Disorders | ICD-10 | F12 |
| **Exclusion** |  |  |
| Endoscopy, wrist, surgical, with release of transverse carpal ligament | CPT | 29848 |
| **Matching Criteria** |  |  |
| Anxiety, dissociative, stress related, somatoform and other nonpsychotic disorders | ICD-10 | F40-F48 |
| Morbid Obesity | ICD-10 | E66.01 |
| Opioid Analgesics | VA | CN101 |
| Emergency Department Services | CPT | 99281, 99282, 99283, 99284, 99285, 99288 |
| **Outcomes** |  |  |
| Opioid Analgesics | VA | CN101 |
| tramadol | RxNorm | 10689 |
| hydromorphone | RxNorm | 3423 |
| hydrocodone | RxNorm | 5489 |
| fentanyl | RxNorm | 4337 |
| oxycodone | RxNorm | 7804 |
| Postoperative Follow-up | CPT | 99024 |
| Emergency Department Services | CPT | 99281, 99282, 99283, 99284, 99285, 99288 |
| **Other Opioids** |  |  |
| buprenorphine | RxNorm | 1819 |
| Butorphanol | RxNorm | 1841 |
| butalbital | RxNorm | 19860 |
| benzyhyrdocodone | RxNorm | 2001352 |
| dezocine | RxNorm | 22713 |
| dihydrocodeine | RxNorm | 23088 |
| sufentanil | RxNorm | 56795 |
| alphaprodine | RxNorm | 594 |
| levorphanol | RxNorm | 6378 |
| methadone | RxNorm | 6813 |
| nalbuphine | RxNorm | 7238 |
| remifentanil | RxNorm | 73032 |
| oxymorphone | RxNorm | 7814 |
| tapentadol | RxNorm | 787390 |
| pentazocine | RxNorm | 8001 |
| Codeine | RxNorm | 2670 |
| Morphine | RxNorm | 7052 |
| Meperidine | RxNorm | 6754 |
